# Cold Steel, Weak Flesh: Mechanism, Masculinity and the Anxieties of Late Victorian Empire

**DOI:** 10.1080/14780038.2016.1269538

**Published:** 2017-01-13

**Authors:** Michael Brown

**Affiliations:** ^a^ Department of Humanities, Digby Stuart College, University of Roehampton, London, UK

**Keywords:** Masculinity, gender, war, empire, technology

## Abstract

This article considers the reception and representation of advanced military technology in late nineteenth- and early twentieth-century Britain. It argues that technologies such as the breech-loading rifle and the machine gun existed in an ambiguous relationship with contemporary ideas about martial masculinities and in many cases served to fuel anxieties about the physical prowess of the British soldier. In turn, these anxieties encouraged a preoccupation in both military and popular domains with that most visceral of weapons, the bayonet, an obsession which was to have profound consequences for British military thinking at the dawn of the First World War.

‘Ye Tories, say what should be done;

What would you like to do?

What man, machine, or Maxim gun,

Can pull Old England through?

They only shake the solemn head,

And parrot like reply,

“*The gilt is off the gingerbread,*

*The bloom is off the rye*!”’

*Punch*, 11 July 1885

## Introduction

In George Orwell’s *The Road to Wigan Pier* (1937) the author recalled attending George V’s funeral the preceding year and being taken aback by the appearance of the crowd. ‘It was impossible’, he claimed, ‘not to be struck by the physical degeneracy of modern England … Hardly a well-built man … and not a fresh complexion anywhere’. Even the Guards accompanying the coffin ‘were not what they used to be’:

Where are the monstrous men with chests like barrels and moustaches like the wings of eagles who strode across my childhood’s gaze twenty or thirty years ago? Buried, I suppose, in the Flanders mud … If the English physique has declined, this is no doubt partly due to the fact that the Great War carefully selected the million best men in England and slaughtered them, largely before they had had time to breed. But the process must have begun earlier than that, and it must be due ultimately to unhealthy ways of living, i.e. to industrialism. I don’t mean the habit of living in towns – probably the town is healthier than the country in many ways – but the modern industrial technique that provides you with cheap substitutes for everything. We may find in the long run that tinned food is a deadlier weapon than the machine gun.^1^

Orwell’s lament links war, eugenics and nutrition in a powerful expression of declinist anxiety, but he was far from novel in voicing such concerns. Indeed, it is remarkable how commonplace this passage seems in historical retrospect. Notwithstanding the catastrophic social and psychological impact of the First World War, or indeed Orwell’s own imagined memories of a lost Edwardian age of barrel-chested Guardsmen, these comments would not have seemed out of place thirty, forty, perhaps even fifty, years earlier. As Orwell himself suggested, the process of national physical decline was widely held to have begun with the advent of urban industrialism. Though the precise targets had changed – cheap tinned food was not so much the concern of later nineteenth-century reformers as urbanism itself – the tenor and tone of the comments were much the same.

What is also intriguing about Orwell’s statement is the way in which he links tinned food with the machine gun. That terrible weapon, which wrought so much destruction on men’s bodies on the Western Front, is compared to the humble tin of corned beef which, in Orwell’s opinion, wreaks perhaps even greater havoc on the physiques of the malnourished working classes. Both technologies are here presented as the malign products of industrial modernity and both are seen to have powerful implications for masculinity. Moreover, in his evocation of a ‘modern industrial technique’ that provides ‘cheap substitutes for everything’, Orwell seems to suggest something about authenticity, or the lack of it, in modernity. Just as the modern abattoir and meat-packing industry has produced nutritionally inadequate pseudo-food, so too has modern society and modern warfare conspired to produce pseudo-men.^2^

This article explores the origins of these anxieties in the later nineteenth century. It suggests that they emerged at the confluence of a variety of powerful social and intellectual forces, namely Social Darwinism, New Imperialism and technological modernity. It focuses in particular on the body of the soldier. As Orwell’s comments suggest, one did not necessarily have to be a tub-thumping jingoist to invest the soldier’s body with deep meaning, or to read off it something profound about the state of the nation and of national manhood. As this article will demonstrate, the soldier’s body was a source of concern for military thinkers for much of the late Victorian period. Indeed, it was at this time, with the rise of popular militarism, that the solider came to be of concern to the nation as a whole.

Historians have long debated the place of war and the military in British culture at the turn of the twentieth century. While Olive Anderson and Anne Summers drew attention to the increased popularity of the army from around the time of the Crimean war (1853–6), others have sought to exculpate Britain from the charge of militarism.^3^ Michael Howard, for example, claims that ‘British writers showed themselves less inclined than were their Teutonic cousins to glorify war as the supreme act of the State … the consciousness of military achievement in the past and the determination if necessary to parallel it in the future, all this was there, but without rancour or fanaticism; still underpinned by a strong Christian ethic and leavened by the values of Victorian liberalism’.^4^ John MacKenzie, meanwhile, detects a ‘paradox of British history’ in the fact that ‘Historians have often seen the British as essentially anti-militarist’ despite the ‘ubiquity of military images … in popular culture in the late nineteenth and early twentieth centuries’.^5^ This ‘pleasure culture of war’ has been explored by a number of scholars, including Michael Paris, whose work supports MacKenzie’s suggestion that Britain was more invested in a culture of war than some might acknowledge.^6^ Indeed, though Summers, like Howard, contrasts the British experience with that of Germany, claiming that ‘Militarism in Britain before the Great War was utterly different from “Prussianism”’, she sees Anglicanism and liberalism not as having a mitigating effect but rather as perpetuating a more extensive culture of bellicose nationalism. Whereas Prussian militarism was a ‘ruling-class ideology’, an ‘instrument of the professional armed forces’, in Britain, she maintains, ‘Liberal, popular and independent forms of militarism flourished alongside and indeed in opposition to militarism in its official forms’. ‘Militarism’, she goes so far as to suggest, was an ‘integral part of the liberal political culture of the country’.^7^

Whatever was the case, there can be little doubt that the latter decades of the nineteenth century saw the army assume an ever greater and more positive role in British society. MacKenzie’s characterization of a complete transformation from the ‘rapacious and licentious soldiery’ of the early century to the soldier hero of the 1880s and 1890s may be somewhat overdrawn but it remains broadly accurate in outline.^8^ This rise in status was due in no small part to the advent of New Imperialism. Beginning with the popular politics of Benjamin Disraeli in the 1870s and reaching its zenith under Joseph Chamberlain’s tenure as Secretary of State for the Colonies (1895–1903), this era witnessed a revived territorial expansionism centred on the North West frontier of India and, more especially, Africa. Between 1874 and 1902 hardly a year would pass without British forces being involved in some form of conflict in a far flung colonial outpost. The increased physical and imaginative distance at which the army now operated tended to obviate established associations between the army and domestic political tyranny, allowing it to attain the level of popular support that the navy had long enjoyed.^9^ Meanwhile, the *relative* ease with which these conflicts were resolved allowed the public to indulge their fantasies of conquest and adventure without undue anxiety. Colonial conflict, according to MacKenzie, presented ‘the image of war, without its guilt and only five-and-twenty per cent of the danger’.^10^

What is more, by the latter decades of the nineteenth century, war itself had come to occupy an important place within contemporary social and political thought. As Darwinian concepts of selection and competition in the animal kingdom were transformed through the writings of Herbert Spencer and others into social forms which emphasized rivalry and conflict between humans, war came to be seen by some as the ultimate expression of collective human endeavour.^11^ Developing alongside New Imperialism, such competition and conflict quickly assumed dramatic implications.^12^ As rivalry between the major European powers intensified in the face of economic depression and dwindling resources, nation states were naturalized into ‘races’ and the struggle for ultimate supremacy assumed the imagined form of an all-out war of annihilation.^13^ Of course, such racialized discourses had profound implications for the representation of colonial conflict. For some, wars of conquest against supposedly uncivilized ‘savages’ had a moral quality which set them apart from the horrific spectre of European conflict.^14^ For others, notably the biometrician and eugenicist Karl Pearson, they functioned as a form of preparatory group calisthenics, allowing the nation to be ‘kept up to a high pitch of external efficiency … by way of war with inferior races’.^15^

Yet at precisely the moment when the physical, moral and intellectual fitness of the ‘national stock’ assumed paramount importance in the struggle for survival, European society was wracked by profound concerns about racial degeneration and the enervating effects of modern industrial society.^16^ ‘It was a time’, according to Paul Crook, ‘when strident declamations of Western superiority and faith in inevitable progress were combined with widespread evidence of disenchantment and doubt’.^17^ Such degenerative discourses played out differently in different national contexts, but in Britain, as we have seen, they were especially linked to fears of imperial and national decline. In large part these fears centred on the physical capacities of the British population and on the ability of the British male to defend and sustain the imperial project. While many historians have placed particular emphasis upon the indifferent performance of the British army in the Second South African War (1899–1902),^18^ the reality is that, in military circles at least, these anxieties went back much further, to at least the early 1870s.^19^ Nevertheless, the war certainly galvanized popular and governmental concern, with the establishment of the Inter-Departmental Committee on Physical Deterioration and the publication of numerous tracts bemoaning the state of British manhood and military preparedness. Explanations and theories varied between authors but certain key elements remained consistent. The journalist Arnold White’s *Efficiency and Empire* (1901), for example, was fairly typical in locating the physical enfeeblement of the British race in the decline of rural life and the rise of industrial urbanism.^20^

There is an essential irony here. If colonial conflict was to function, as in Pearson’s imagination, as a kind of advanced training for the race, then why, after decades of such warfare, had British soldiery not reached a peak of physical efficiency? The reality was that colonial wars were seldom, if ever, won by feats of physical prowess. Rather, and as many contemporaries were well aware, they were won by superior technology, by the army’s possession of weapons such as breech-loading rifles, machine-guns and quick firing artillery that decimated the ranks of their lightly armed opponents.^21^ To be sure, Social Darwinists would point to such technological advantages as evidence of British racial superiority. And yet at the same time, those arms were the product of a system of industrial manufacture which supposedly threatened the very physical integrity of the race.^22^

This article highlights the ironies and ambiguities of Social Darwinism and New Imperialism through an exploration of the reception and representation of advanced military technology in late nineteenth- and early twentieth-century Britain. There is a substantial body of literature on the development of British military strategy in the later nineteenth and early twentieth centuries. While some scholars emphasize innovations in British military doctrine, others assume a general opposition within the military hierarchy to the adoption of new technology, an opposition based upon an aristocratic rejection of industrial modernity and an attachment to military tradition.^23^ This article will demonstrate how the reception of technologies such as the machine gun was rather more complex and ambivalent than this latter position suggests. Moreover, it will explore these complexities in relation to late nineteenth-century anxieties about masculinity and will demonstrate how they emerged out of the specific context of colonial warfare. Britain’s small wars of empire produced a wide range of attitudes towards the metropolitan self and colonial other, but the image of the pigeon-chested, slum-dwelling ‘Tommy’ obliterating the muscular ‘noble savage’ with a weapon no more distinguished than a power loom posed troubling questions about the moral and physical qualities of the British race. In turn, these anxieties helped to shape a preoccupation in both military and popular domains with that most visceral of weapons, the bayonet, an obsession which was to have profound consequences for British military thinking at the dawn of the First World War.^24^

## Cardwell’s Army: Bantams and Boy Soldiers

In February 1874, Leith Adams, Surgeon-Major of the London Recruiting District, presented a paper to the Royal United Services Institute (hereafter RUSI) on ‘The Recruiting Question, Considered from a Military and Medical Point of View’. His gloomy view was that ‘ever since the Crimean war, there has been a steady falling off in the recruiting market, not only as regards the supply, but the physical capacities, of the men’. Once, he claimed, a recruiting sergeant might have entered any town or village in Britain and found numerous stout men suitable for the army. Back then, ‘a few smart lads of 5 feet 7 inches in height’ were all that was required: ‘now we are in need of a much lower standard and cannot get them’.^25^ While he acknowledged that average height was not the only factor for the authorities to consider, he nonetheless maintained that the general standard of recruits was in steep decline. Of especial concern was the increasing number of recruits of less than twenty years old, many of whom were physically enfeebled ‘striplings’, members of that ‘unimprovable, stunted and staminaless race met with in crowded cities’. ^26^

Such concerns were not especially new. Military leaders were always apt to romanticize the past and to suggest that they did not make soldiers like they used to. Indeed, while Adams and others might have looked back to the Napoleonic era as one in which all recruits were strapping country lads with the makings of Guardsmen, the Duke of Wellington had famously described his army as the ‘scum of the earth’.^27^ In the 1870s, however, a number of factors combined to lend these perennial misgivings a particular potency. For one thing, the startling success of the Prussian-led German coalition, which annihilated the forces of the French Second Empire and Third Republic in 1870–1, had sent shockwaves through the British military and political establishment. Together with Prussia’s defeat of the Austrian Empire in 1866, this remarkable victory convinced many that the equilibrium of power on the Continent had shifted, due in no small part to Prussia’s superior military leadership, organization and use of technologies such as railways, artillery and breech-loading small arms. Though Britain was not directly threatened by the conflict, the crushing defeat of France can be said to have initiated a spirit of declinism which would haunt British military and political thinking for the rest of the century and beyond. As one commentator put it:

We have learned this – that we in England are not what we were, that others have outstripped us in the race, and taken from us that place of honour taught as a Briton’s birthright; and this is not because we are worse than our fathers, for we are better; better in science, better in commerce, better in war, but because we are not the best, as they were.^28^

In some quarters of the military, Prussia’s success encouraged a spirit of emulation. In 1878, for example, British soldiers were issued a Home Service Helmet more than a little reminiscent of the distinctive Prussian *Pickelhaube*. At a more profound level, some called for the adoption of Prussian tactics and forms of organization, most notably (and controversially) the establishment of universal conscription.^29^

In actual fact, by the early 1870s the British army had already undergone a substantial reorganization. In 1858, in the aftermath of the administrative debacle of the Crimean War, the government had established a Royal Commission to consider army reform. Momentum had stalled in the face of opposition from the East India Company and military conservatives, but when William Gladstone became prime minister in 1868 he gave Edward Cardwell, Secretary of State for War, the political authority to complete the task. The result was a series of Acts passed between 1868 and 1871 which effectively created a ‘New Army’.^30^ In order to integrate the army more fully into society, regiments were tied to localities rather than being numbered according to their seniority.^31^ To encourage promotion by merit and undermine the ‘gentlemanly’ ethos of the officer corps, the sale of commissions was abolished, and to alleviate the conditions of service, flogging was outlawed as a peacetime punishment, albeit against near universal opposition from the military establishment. However, perhaps the most important reform, and the one which most directly addressed the question of manpower, was the introduction of ‘short service’.

From the end of the Napoleonic wars, men had conventionally joined up for a period of twenty-one years, though the minimum term of service had been reduced to twelve in 1847. This produced an army of experienced veterans, many of whom re-enlisted, but the overall number of recruits remained low, meaning that Britain lacked a large pool of trained men in the event of a major conflict. Taking his inspiration from the Prussian model, Cardwell therefore created a system of reserve and introduced a shortened period of service. Men could still enlist for up to twelve years but it was now possible to serve as little as six in the regular army so long as they agreed to join the reserves in exchange for reduced pay, an annual period of training and an obligation to serve if required.^32^

Almost as soon as it was introduced, however, short service came under intense criticism for failing to produce the right kind of recruit. Sir Edward Sullivan commented that while it was

the universal desire of the country to possess an efficient army, it does certainly appear strange that Mr .Cardwell’s first step in that direction should be to disorganize and emasculate the small force we already possess … our army has been so rapidly filled with wretched boys of seventeen to twenty, that in some regiments they already number over 50 per cent!

Under the guise of reform, he lamented, ‘we were in fact filling [the army’s] ranks with “starvelings” – with boys ill-fed, ill-grown, and whose muscles and bones are not actually matured’.^33^ Indeed, so trenchant was the criticism that one military doctor was later to characterize the Cardwell era as the ‘The Boy-Soldier Period’.^34^

Such concerns about ‘boyhood’ were predicated on the notion that youth was a distinct period of physical and mental development. As Roberta Park and J. A. Mangan have suggested, educators of the period were keen to encourage athleticism in boys in order to inculcate moral values and to promote a healthy physical development into manhood.^35^ At this time, however, such opportunities were largely confined to the upper and upper-middle classes in public schools. Working-class children, by contrast, were subject to comparatively little structured intervention, at least prior to the twentieth century.^36^ What physical development they might experience would have to be carefully monitored and remedially administered.^37^ By asking boys to do a man’s job, the post-Cardwell army was thus not simply short-changing the nation in terms of the physical capabilities of its front-line soldiers; it was killing or crippling a generation of adolescents. According to Sullivan, ‘Not only do we subject unformed and immature lads of seventeen and eighteen to a severe course of discipline and drill, but in five or six months we ship them out to the tropics to die like flies, or return invalided as a useless charge to the nation’.^38^ The military hygienist Edmund Parkes stated that ‘the youngest recruit should be twenty or twenty-one’ because ‘the process of ossification had not even begun to be completed at the age of eighteen’. He cited examples of young soldiers in whom, during post-mortem investigation, it was found that ‘the ends of the long bones of the legs and arms still showed the cartilaginous state, being incompletely knit’.^39^

For some commentators the issue of manpower was essentially one of political economy. Sullivan argued that ‘In England you will always get what you want at the market price of the article; if we offer the fair wages of men we shall get enough … if we offer the wages of boys we shall get boys and not men’.^40^ With greater opportunities in industry and abroad, the army and its poor pay and harsh working conditions no longer seemed a particularly attractive option to the working classes. ‘The more industry spreads, and the greater the number of its prizes’, claimed one speaker to the RUSI, ‘the smaller is the *residuum* from which recruits can be got, and the worse, physically, morally and intellectually does that *residuum* become’.^41^ According to Frederick Roberts, one of late Victorian Britain’s leading officers, the solution was to make the army more attractive, not by reducing the length of enlistment as Cardwell had done, but by increasing its prestige, lessening the burdens of service and providing opportunities for education and training that would enhance the ex-serviceman’s chances of gainful employment.^42^

For others, however, the issue of ‘boyhood’ had a broader metaphorical resonance. The reason that the army could not get the men it needed was not simply because it was not paying enough but because there were none to be had. For Leith Adams, the poor state of recruits was indicative of a much more intractable crisis of masculinity:

The most dismal and desperate view of the aspect of our Army at the present time is that which associates the physical degeneration of the solider with a general falling off in the strength and stamina of the population. Thus there is the impression that the British soldier is no longer the man he was, and … it is also asserted that the evil portends the coming decline, not only of our military renown, but is a sign of national decay.^43^

To most observers, the cause of this crisis was clear, albeit paradoxical. Evoking the ‘Condition of England’ debates of the previous generation, military thinkers increasingly suggested that, while industry and commerce were the backbone of British power, they might also be the undoing of her military prowess. Industrialization and urbanization had produced an enfeebled race unsuited to the demands of empire. According to Garnett Wolseley, future Commander-in-Chief of the British army:

You have only to visit the great manufacturing centres of England … and to stand outside any great manufactory and see the men and women, boys and girls, coming out at the end of their day’s work, in order to arrive at the same conclusion that I have often done … that they were the most miserable specimens of humanity: and what is most to be deplored is that they are going from bad to worse. At the present day, the people working in our mills and great manufactories throughout England are decidedly inferior as a race physically, to what they were in past times.^44^

Neither was it merely the working classes who were in decline. The 1870s saw a spate of papers delivered to the RUSI on the demise of empires in which parallels were drawn between Britain and ancient Rome. What these speakers suggested was that commercial culture was inimical to military vigour. Some argued that the intelligent middle classes were now inclined to shun military careers in favour of the ‘exciting battle of civilian life’.^45^ Others, including the unabashed Social Darwinist, Captain Henry W. L. Hime, maintained that commercialism promoted luxury, effeminacy and weakness among the population as a whole. If history had taught one lesson, he maintained, it was that ‘as the industrial spirit increases, the military spirit decreases, at all periods and in all climes’.^46^

## Redcoats, ‘Savages’ and the ‘Man’ behind the Gun

The irony in all of this, as Captain Hime recognized, was that the standard of British martial masculinity was degenerating ‘at a time when Tactics are undergoing rapid changes and each change demands a corresponding improvement in the quality of the individual soldier’.^47^ Since the 1860s, basic infantry tactics had indeed undergone a transformation, one initiated, in large part, by technological innovation. The rifle, whose grooved barrel allowed for greater accuracy and stopping power than its smoothbore equivalent, was first introduced on a large scale into the British army in the form of the .577 Enfield Pattern 1853 rifle-musket. While this weapon was in the hands of many troops by the time of Britain’s involvement in the Crimea the following year, tactics remained relatively unchanged from the Napoleonic era when infantry had generally fought in close-order formation, standing shoulder-to-shoulder and exchanging volley fire with an enemy who was often as little as 200 yards away. Weight of fire was rarely enough in itself to rout an opponent and so this exchange was usually followed by a bayonet charge designed to drive the enemy from the field.

**Figure 1.**
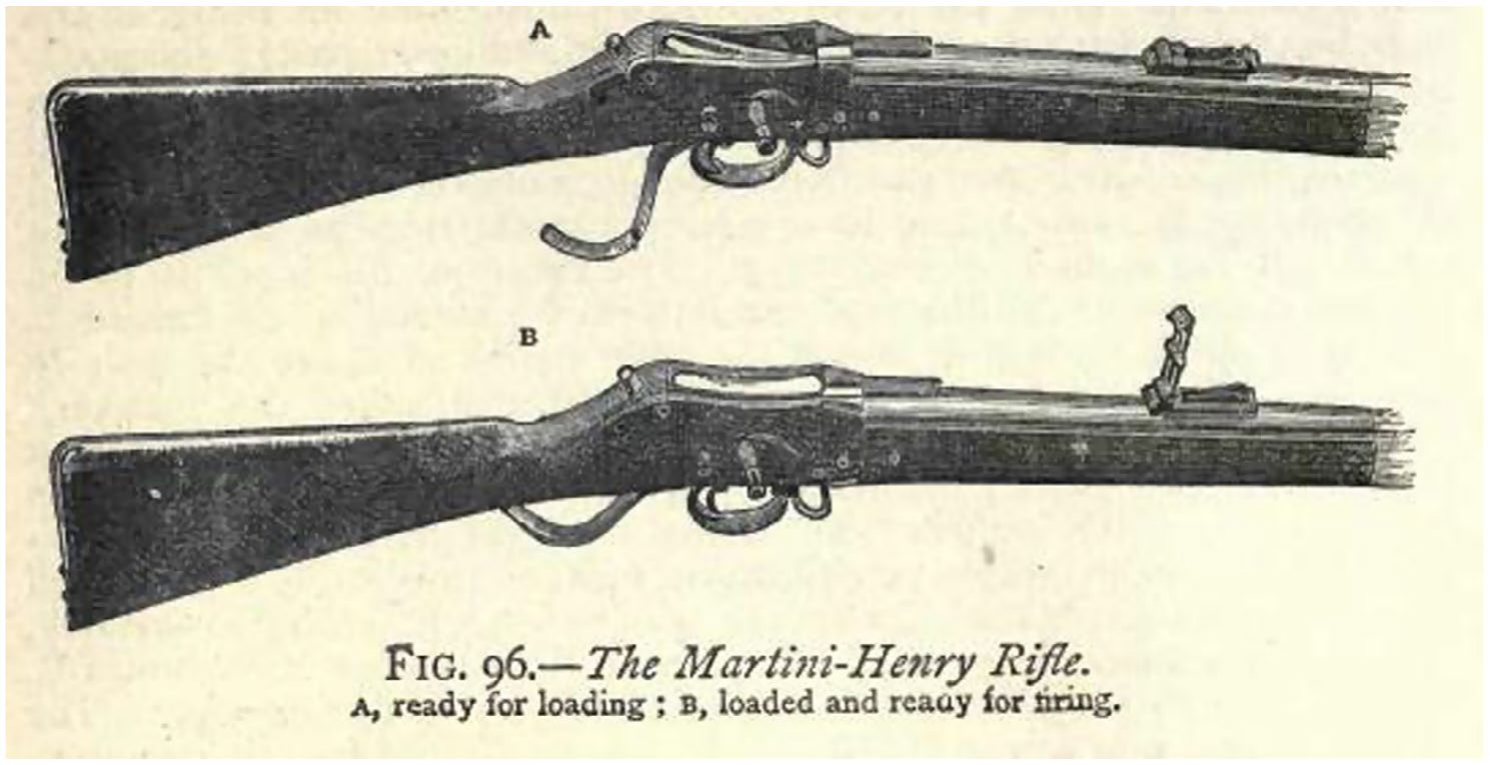
Breech mechanism of the Martini-Henry rifle from R. Routledge, *Discoveries and Inventions of the Nineteenth Century* (London: G. Routledge, 1881), p. 134. Wikipedia Commons, PD-US.

While the experience of the American Civil War (1861–5) demonstrated the power of the new gun, the Enfield rifle was still a muzzle-loading weapon whose operation required a relatively complex drill and which, even in the hands of a well-trained solider, was capable of little more than three aimed shots per minute. In 1866, however, the British introduced the Snider-Enfield, a conversion of the 1853 rifle which, rather than requiring powder and ball to be inserted into the muzzle and pressed home with a ramrod, allowed a single metallic cartridge to be inserted directly into the breech by means of a hinged, locking block. In 1871 this weapon was replaced by the .45 Martini-Henry rifle which, with its single-shot lever-action design, further simplified the process of loading (Figure 1). The trained solider was now capable of firing upwards of 12 aimed shots per minute and, with no need to ram the cartridge into the muzzle, was able to operate the weapon while kneeling or lying down.

**Figure 2.**
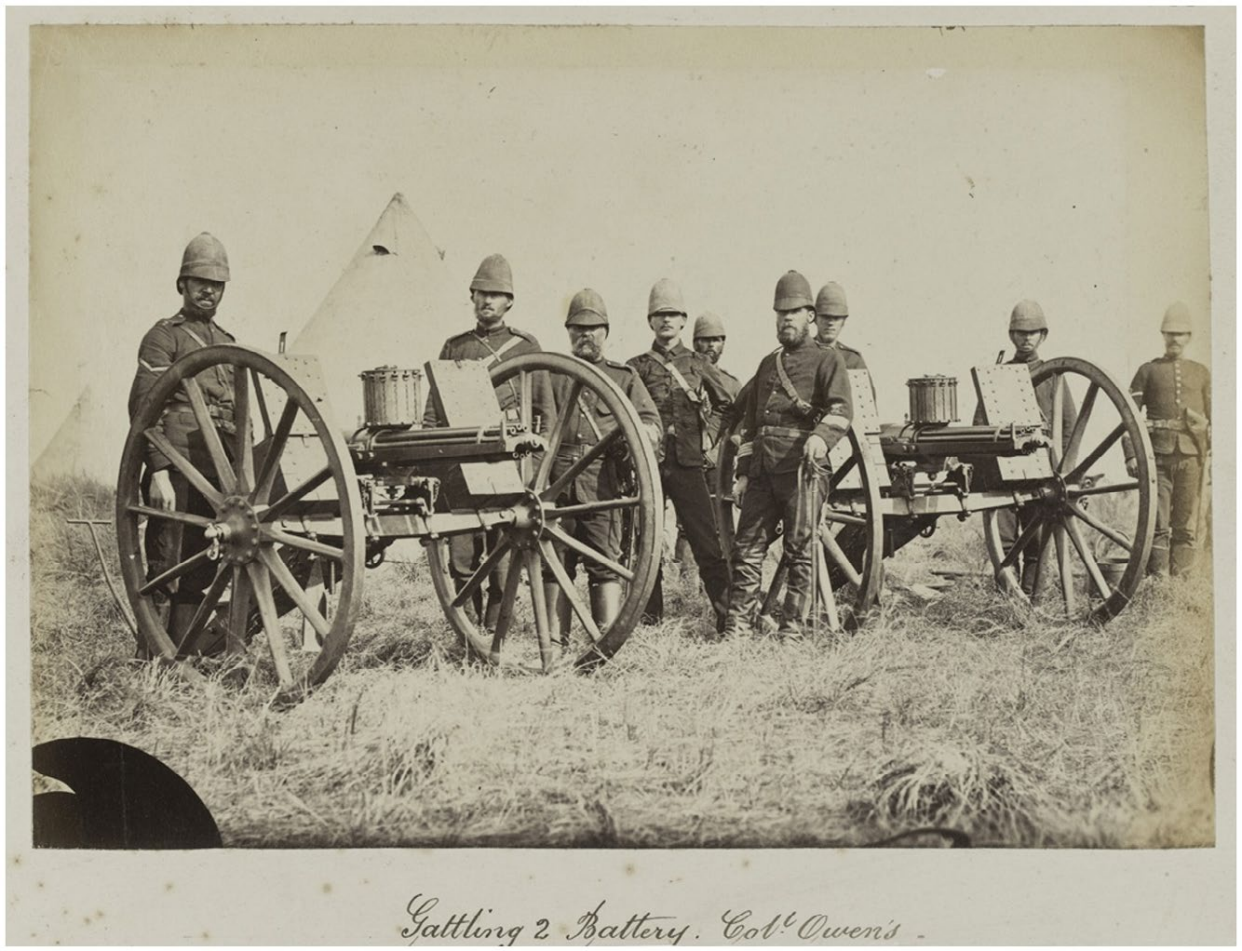
Pair of Gatling guns in service in South Africa, 1879, photograph. Courtesy of the National Army Museum, London (NAM. 1989–10-2–58).

**Figure 3.**
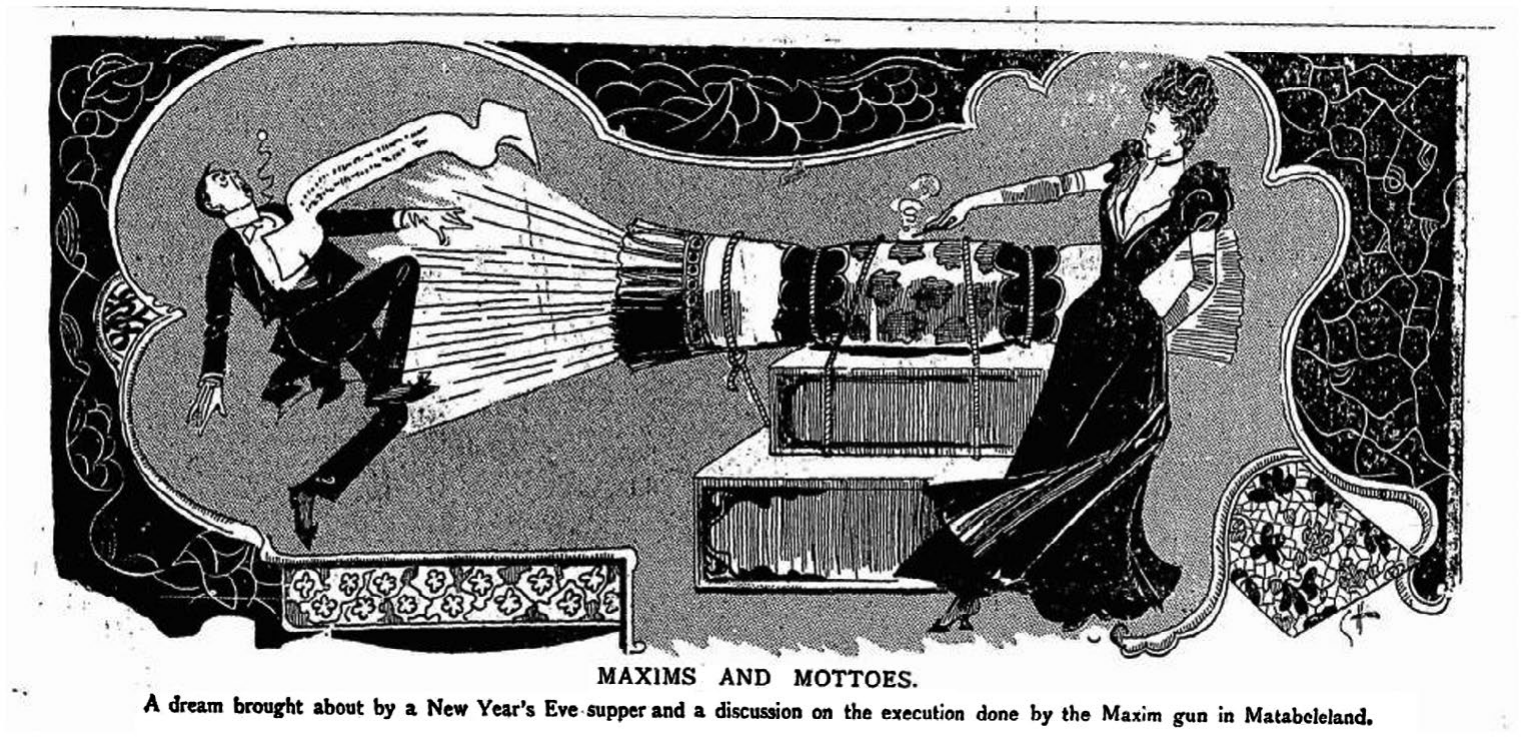
‘Maxims and Mottoes’, *Funny Folks* 6 January 1894, p. 1. © The British Library Board, PENP.NT152.

**Figure 4.**
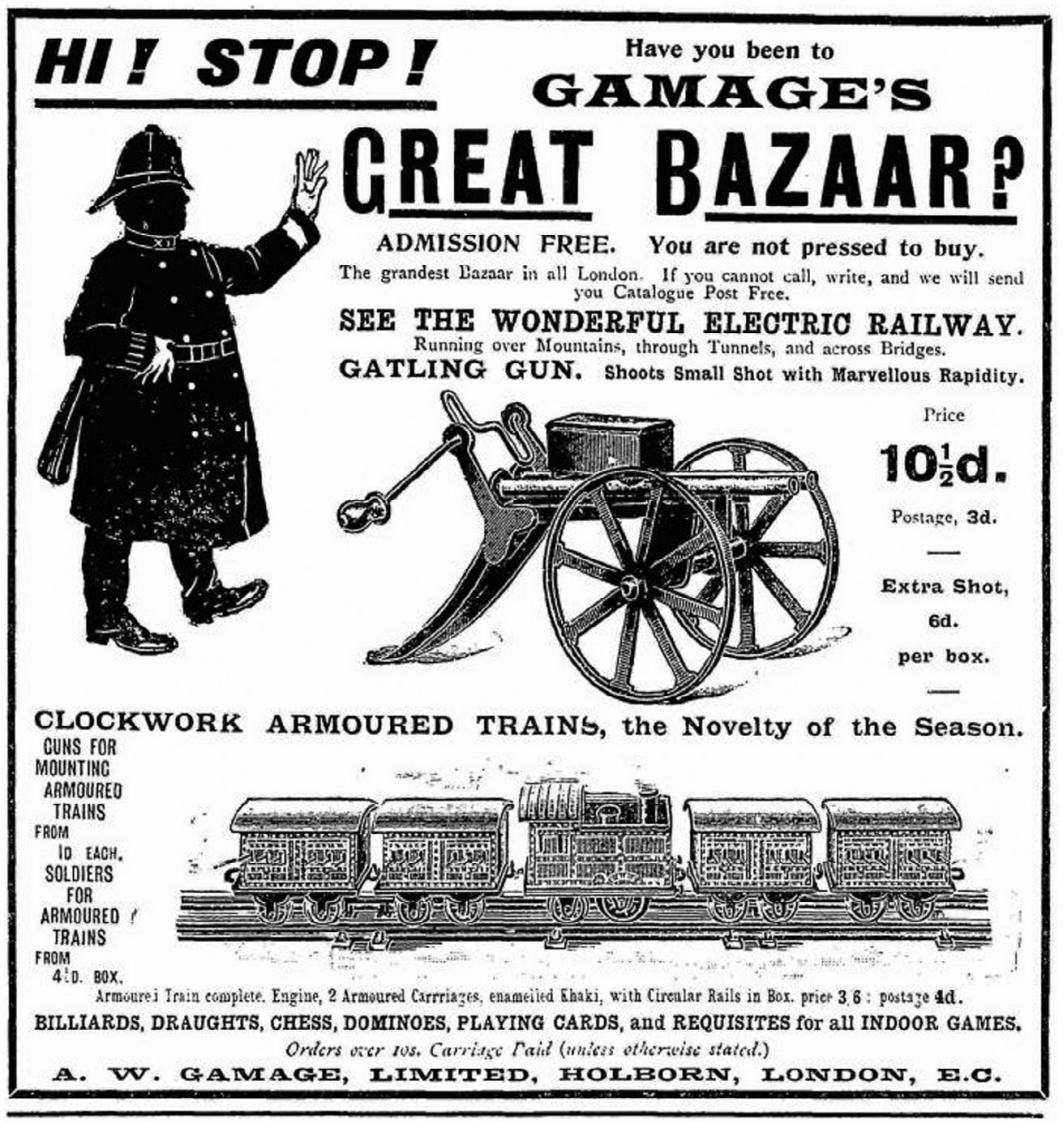
Gamage’s Great Bazaar, *Chums* 19 December 1900, p. 288. © The British Library Board, P.P.5993.rka.

**Figure 5.**
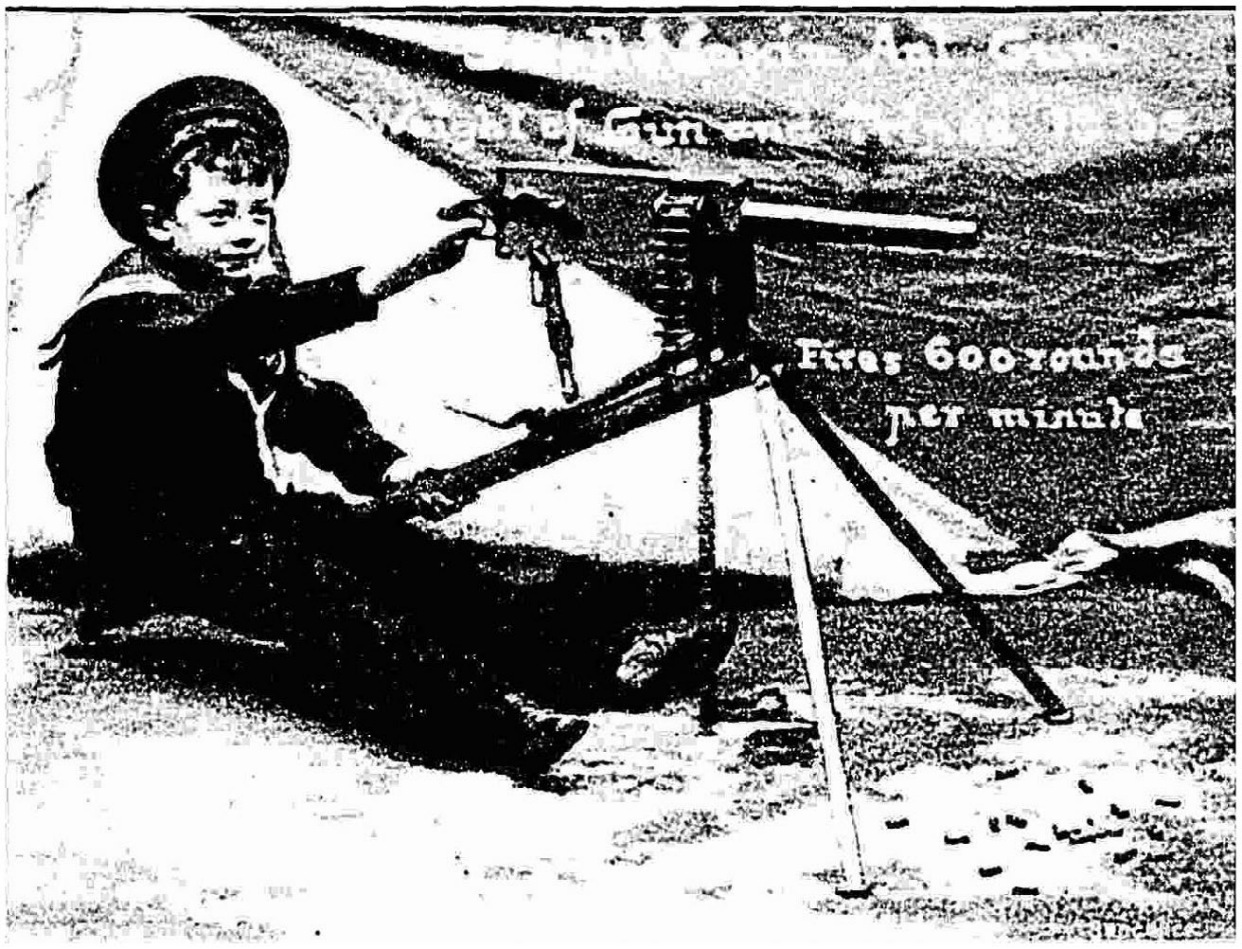
*Hearth and Home*, 4 March 1897, p. 667. © The British Library Board, LOU.LON 385 [1897].

**Figure 6.**
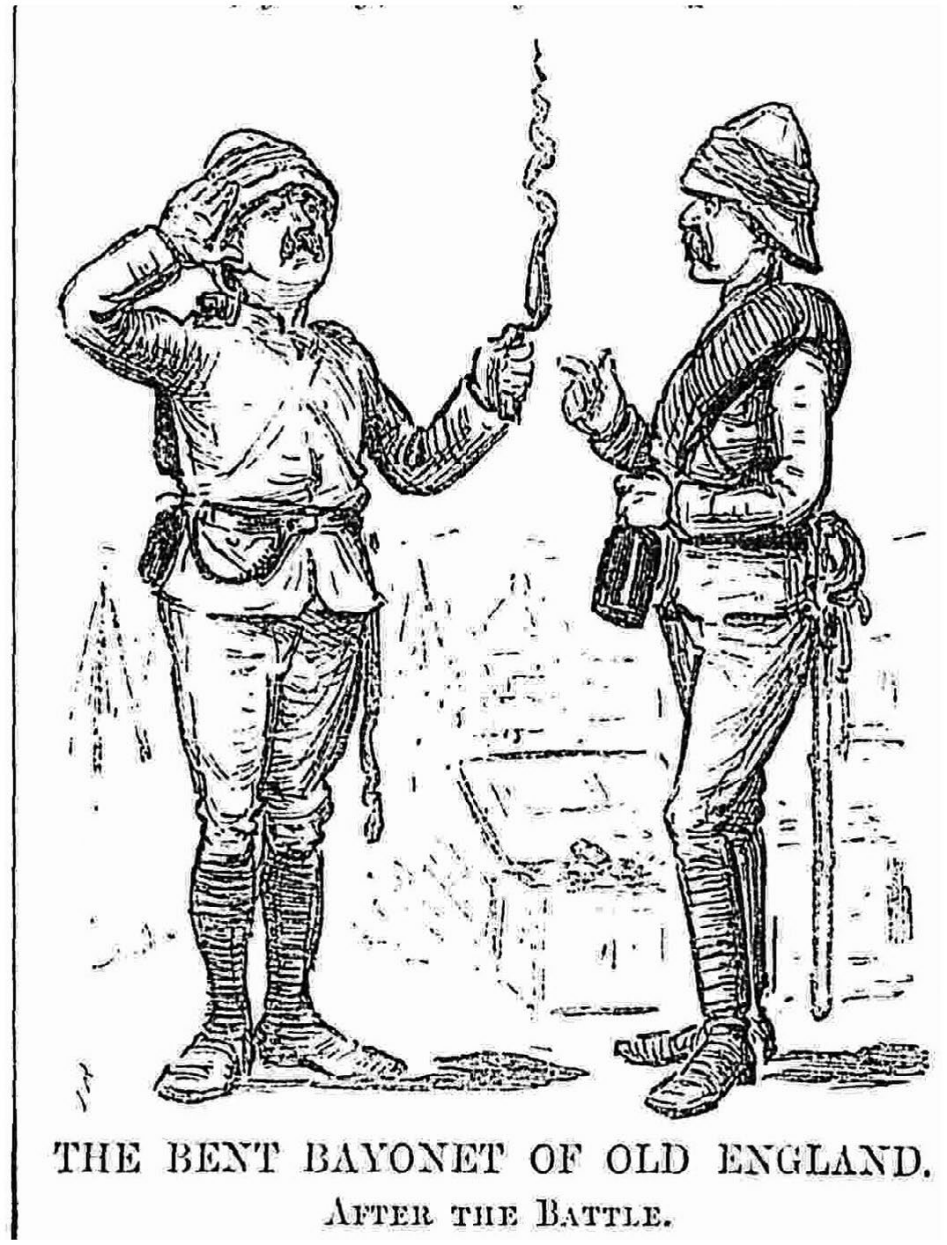
‘The Bent Bayonet of Old England’, *Punch* 23 January 1886, p. 48. © The British Library Board, P.P.5270.

**Figure 7.**
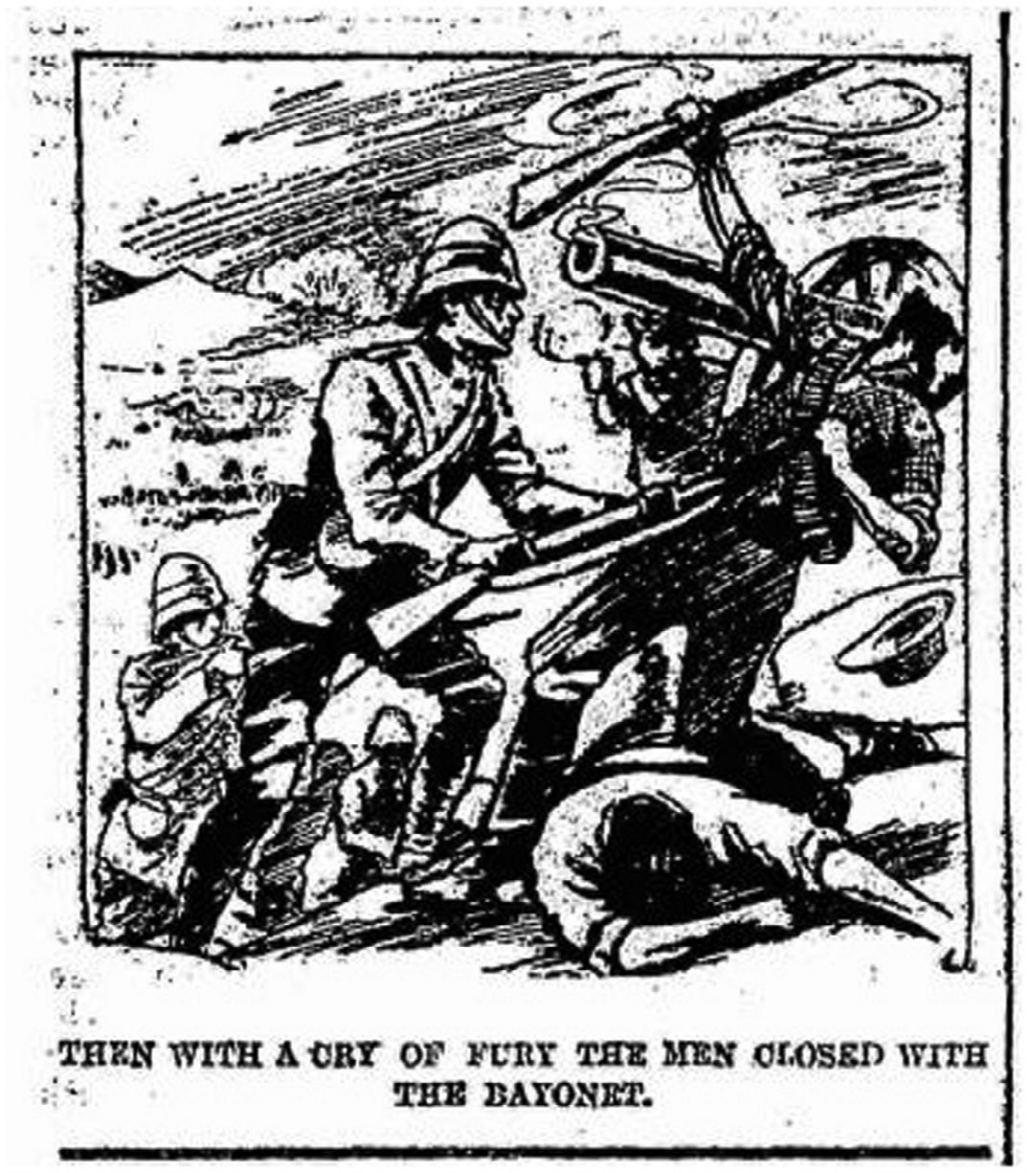
‘Then with a cry of fury the men closed with the bayonet’, *Illustrated Chips* 27 January 1900, p. 2. © The British Library Board, PENP.NT160.

It was the introduction of the breech-loader that truly transformed infantry tactics. The rifle was now a battle-winning weapon in its own right.^48^ Rapid and accurate fire over much longer ranges, potentially from concealed positions, brought an end to the close-order tactics of the musket era and initiated a much looser, decentralized formation.^49^ In turn, as firepower now depended upon individual marksmanship as opposed to half-aimed collective volleys, the skill and resourcefulness of the solider became of paramount concern. The same was true of the ‘moral’ dimensions of the battlefield. Under the old close-order system men had stood side by side under the influence of their fellows and the control of their officers. Now they were expected to display greater initiative and self-control.^50^ In the words of the author of the *Thinking Bayonet* (1871):

We can now in fact allow our soldiers some amount of individuality, of will; free them from some of the iron bands that tether them so tightly; from the incessant alignments, the dressings, the toe-the-line, chins-up business, and let each man carry with him into the greatest ordeal ever invented, some of these higher qualities that make him something more than the beasts.^51^

The introduction of ever more sophisticated mechanical contrivances into the military realm gave rise to a paradoxical language. For some, like this author, the breech-loader was ‘the arm for a man and not a machine’ in that it liberated soldiers from the mindless routines of the Napoleonic era and allowed them to express themselves as intelligent, self-actuating, wilful beings.^52^ For others, however, ‘the application of science to warfare … had the effect of diminishing the value of the individual excellence of the soldier’. According to this observer, ‘the army is becoming more and more like a perfect piece of machinery, in which each separate part works only in its own particular groove’.^53^

Such language exemplifies the anxieties surrounding technology and masculinity that coloured military thinking in the 1870s. Some thought that technology contributed to a decline in martial prowess, others that it made the quality of the solider an even more pressing concern but almost all were agreed that something was very seriously wrong. Moreover, by the end of the decade they were to have even more reason to worry. Many military men looked forward with trepidation to an all-out war between European opponents, but Disraeli’s expansionist foreign policy, carried on with greater or lesser enthusiasm by his successors, ensured that, for the moment, Britain’s wars would be relatively small affairs against lightly-armed colonial opponents. Yet while the technological superiority of the British and imperial forces should have suggested a foregone conclusion, the near debacle of the Anglo-Zulu war was to prove otherwise.

The British intervention in Zululand resulted from a desire on the part of Lord Carnarvon, Secretary of State for the Colonies, and Bartle Frere, High Commissioner of Southern Africa, to unite the patchwork of territories in the region into a formal confederation under British jurisdiction. In 1877, the Boer Transvaal Republic had been annexed, leading to increased tension along the border with the Kingdom of Zululand. Without consulting the British government, Frere decided to neutralize the Zulu threat by issuing an ultimatum to their leader, King Cetshwayo. When he inevitably refused, Frere ordered an invasion.^54^

The British forces, led by Lord Chelmsford, entered Zululand in January 1879, fully confident of success. However, soon after crossing the border they experienced one of the worst disasters in British military history as a force of around 20,000 Zulu warriors, mostly armed with little more than cows-hide shields and short spears or *assegai*, overran and destroyed a portion of the British main column encamped at Isandlwana. Over 1,800 troops were killed, including nearly 800 British regulars. Unable to form up in defensive order they had been no match for the Zulus at close quarters.

The British media reacted to this humiliating defeat by trying to extract whatever honour they could from the disaster and the fate of the men of the 24th Foot was soon immortalized in grandiloquent verse and heroic art.^55^ The subsequent defence of Rorke’s Drift by around 150 British and colonial troops against around 3,000 Zulus also provided a salve to wounded British pride and resulted in the award of an almost unseemly number of Victoria Crosses, Britain’s highest award for valour.^56^

In spite of eventual victory, the experience of the Zulu war posed troubling questions about the comparative merits of the combatants. As Catherine Anderson has demonstrated, representations of the Zulu within British popular culture varied considerably between those produced during the course of war and those produced after it, a fact neatly satirized by *Funny Folks*, who contrasted an early characterization of the Zulu as having an ‘intellect considerably below that of a chimpanzee’ and as being ‘the scum of the African continent’ with a later one which praised their military acumen and described them as ‘models of manly beauty’.^57^ Certainly, the years following the end of the war saw a raft of travelogues and other publications in which the Zulu was cast as the epitome of the noble savage. Observers looked admiringly upon their social relations, their warrior culture and, in particular, their physical form.^58^ ‘The physique of the men’, wrote one Royal Artillery officer, ‘is the perfection of manly strength and symmetry. Tall, muscular and well-knit … they irresistibly recall the sculptured attitudes of athletes in a Roman circus’.^59^ Anderson has suggested that, in the warrior ethos of the Zulus, British observers found a mirror of their own increasingly militarized masculinity.^60^ But in actual fact, the valorization of the Zulu warrior often came, either explicitly or implicitly, at the expense of his British conqueror. In all its ‘primitive’ simplicity and vitality, Zulu society was everything that urbanized, commercialized and industrialized Britain could not be. It was hardly a surprise, therefore, that the enfeebled boy-soldier of the British army was no match for his Zulu opponent. As Garnett Wolseley put it, ‘To put the ordinary Tommy Atkins reared in Whitechapel, against the hillside savage warrior is not only the worst of folly … it is cruel to the man concerned and criminal to the nation’.^61^

This mismatch in martial manliness was epitomized by the inability of the British soldier to ‘get to grips’ with his opponent and best him in one-on-one, hand-to-hand combat. In a letter to a colleague, for example, Frederick Roberts confessed that ‘nothing, perhaps, tries British soldiers more than a sudden rush by a determined body of men, who mean to die, such as the natives of South Africa ... The fact is, our men are not used to men coming at them, and are somewhat taken aback when they do’.^62^ As a result, during both the Zulu war and Sudanese campaign (1884–5), British troops were forced to abandon modern tactics and revert to a close-order square formation so that they might keep the enemy at bay by volume of fire from their Martini-Henrys.^63^ Practical though it may have been, many saw this as an ignominious development, implying, in the words of one author, ‘a sense of inferiority in personal prowess’.^64^ Ever the realist, Wolseley had no such qualms. ‘It is only by our superior arms and superior discipline that we can beat these fighting tribes who have done soldiers’ work since childhood’, he admitted. ‘If we meet them with the same tactics with which we should meet a French army, we are almost certain to be defeated’.^65^

Of course, no one was suggesting that British soldiers should abandon the breech-loading rifle in favour of the *assegai* merely to prove their worth as men, but the notion that the security of the empire rested upon technological advantage more than inherent martial prowess served to fuel those anxieties that bubbled away below the surface of jingoistic bombast. Scholars such as Graham Dawson and Michael Paris have shown how late nineteenth-century juvenile literature inculcated young boys into a pleasure culture of war, an imagined realm of rugged imperial masculinities.^66^ However, when read against the grain, such narratives can reveal deeper anxieties and ambiguities. Take George Manville Fenn’s ‘Off to the Wilds’, which was serialized in G. A. Henty’s boy’s paper *Union Jack* in the winter of 1881–2. Written in the immediate aftermath of the Anglo-Zulu war it is tale of two young brothers, Dick and Jack, at large in the wilds of southern Africa. At one point, laden down with the carcass of a recently slain antelope, they are surprised by ‘half a dozen blacks, armed with assegais and clubs’ who advance on them with ‘fierce and threatening gestures’. ‘Shall we offer them our guns and ammunition if they will let us go?’, asks Jack. ‘No’, replies Dick, ‘If we do that they will strip us to the skin’. He suggests they must ‘Show fight’. ‘But they are big fighting men and we are only boys’ counters Jack. ‘But we are English boys and they are only savages’ Dick responds, ‘so come along’. The boys proceed to push through the group of warriors, their own black companions crying ‘Let my lords the big-lion killers with their wonder-guns come by’. Bowed by this spectacle, the warriors drop their façade of menace and, pleading starvation, beg for some of the boys’ meat, a favour which, with paternalistic largesse, these so-called ‘young kings’ duly grant them.^67^ Now, at one level this is an eminently generic tale of racial superiority, the kind which suggests, in the words of Michael Paris, that the ‘inherited prowess’ of the Anglo-Saxon was such that ‘even a British boy could successfully take on an enemy warrior and win’.^68^ Read against the backdrop of the recruiting crisis of the 1870s, however, it takes on a subtly different significance. After all, it is clear that in this story the boys’ authority derives solely from their possession of a rifle, and while this rifle makes boys of African men, it also suggests the problematic possibility that it makes pseudo-men of British boys.

## The Machine Gun: Technology, Efficiency and Masculinity

Significant though the breech-loading rifle undoubtedly was, the technology which did most to focus anxieties about masculinity and empire was the machine gun. The idea of a weapon which could fire multiple projectiles without the need for reloading after each shot was as old as the firearm itself, but it was not until 1862 and the advent of the Gatling gun that it was fully realized. Dr .Richard Jordan Gatling (1818–1903) was an American inventor whose weapon consisted of between six and ten barrels grouped around a central shaft, each of which had its own breech and locking mechanism. The shaft was turned by means of a hand crank and as the barrels rotated they would receive a cartridge from a hopper located on the top of the weapon. A cam would then engage to lock the cartridge in the barrel before releasing a striker to fire it. In principle, the weapon could thus be kept firing so long as it was supplied with ammunition and as quickly as the handle was turned, up to a maximum of around 200 rounds per minute.^69^

In his pioneering history of the machine gun, John Ellis claims that the British army reacted to this new technology with ‘unremitting hostility’ and that this ‘was not a rational response to either technical or financial considerations’ but was ‘rooted in the traditions of an anachronistic officer corps’.^70^ Certainly some military men did express scepticism over its merits and the relatively poor performance of the French *mitrailleuse* volley gun in the Franco-Prussian war did little to change their opinion.^71^ By contrast, the navy, who saw the potential of the machine gun for shipboard defence, were more enthusiastic, and, after protracted trials, the Gatling was officially adopted by the navy in 1871 and the army in 1874. Moreover, while military strategists argued over the role of the machine gun in any potential European conflict, the weapon was soon utilized in Britain’s ‘small wars’ of empire.^72^ It was first employed in the Third Anglo-Ashanti War (1873–4) and, later, four Gatlings under the command of the Naval Brigade accompanied Lord Chelmsford on his second invasion of Zululand in May 1879; two of these were put into action at the decisive battle of Ulundi where, according to Lord Chelmsford himself, they ‘did very good service indeed, as was shown afterwards by the number of dead Zulus found on the ground’ (Figure 2).^73^

Indeed, many military theorists, even those who were relatively sceptical about its application in the European theatre, thought the weapon especially well-suited to colonial warfare. Of particular importance in this context was what was known, euphemistically, as its ‘moral effect’, that is, its capacity to terrify and ‘pacify’ a technologically less sophisticated people. As Sir Percy Douglas, former governor of the Cape Colony, put it:

On the question of the use of the Gatling gun and its applicability to warfare against uncivilized people, from my experience at the Cape, I can say it would be very valuable. I have no doubt one gun of that sort, with a very few men, would hold a post against any number of natives. It would have this disadvantage, that it makes such a horrible row in going off that it would frighten the foe away; whereas you want them to stand so as to get hold of them. It would no doubt strike great terror into these people. The only thing I am afraid off is that none of them would stop.^74^

As this quotation reveals, the other key point in favour of the weapon was its capacity to maximize firepower without increasing manpower. In many ways this was in line with Gatling’s own thinking. His invention was conceived against the immediate backdrop of the American Civil War and he later claimed that he had been inspired by a desire to lessen the number of troops required in battle and hence the number of casualties. Armed with one of his guns, he maintained, one man ‘might do as much battle duty as a hundred’.^75^ From its very inception, therefore, the machine gun was conceived as a model of industrial efficiency. In the context of late nineteenth-century British concerns about the quality and prowess of its soldiery, however, it possessed even greater significance. Major Hale of the Royal Engineers was not alone in regarding the Gatling as a potential answer to the ‘recruiting question’. ‘I see it as ten nerveless soldiers’ he claimed, and it is best employed ‘at that point when your own men’s nerves begin to fail’.^76^ With ironic circularity, then, the solution to the morally and physically degenerative consequences of industrial modernity was to be found in machines. In reality, however, men’s nerves continued to get in the way of efficient, mechanized killing. The Gatling gun was a comparatively delicate weapon and in the heat of battle its anxious operators had a tendency to over-crank the firing handle, overheating the barrel and resulting in stoppages. According to one commentator, an embodied expertise was what was required:

The men set to work machine-guns should be as intimately acquainted with every part of the mechanism as a surgeon is with the anatomy of the human body. And just as the latter can tell by his patient’s pulse what is wrong with his anatomical system, so should the man working a machine-gun be able to tell instantaneously, by the feel of a throb or jerk of the firing lever in his hand, what has gone wrong with the internal mechanism of his piece, and at once be in a position to clear the action without the use of force, thus avoiding accidents, while at once bringing his gun back into action.^77^

For others, however, the answer lay in reducing human input to an absolute minimum. Reflecting on his experiences in the Anglo-Zulu war, Lord Chelmsford suggested that Gatlings were somewhat too complex for his men to operate: ‘if a machine gun can be invented that may safely be entrusted to infantry soldiers to work, and could be fired very much as one grinds an organ, I am satisfied of its great value’.^78^

In 1884 just such a weapon arrived in the shape of the Maxim gun. Designed by expatriate American inventor, Hiram Maxim, this weapon utilized the recoil power of the round being fired to extract the casing and cycle another round into the chamber before firing again. Maxim’s weapon was the quintessential *machine* gun in that it required comparatively little human intervention. So long as the trigger was depressed and it was fed with ammunition it could be kept firing at up to 500 rounds per minute. As Maxim himself stated in an interview with the boy’s paper *Chums*, whereas the Gatling had a tendency to jam when less experienced gunners were ‘confronted by a dangerous enemy’, his weapon used an ‘automatic system’ and ‘as the recoil energy has no nerves, it never gets excited and can always be depended upon to work the gun with mathematical precision’.^79^

As the zenith of efficient, mechanized killing, the Maxim gun acted as the catalyst and focus for a range of contemporary hopes and fears about social, cultural and economic modernity, providing an imaginative and functional link between technologies such as the typewriter and movie camera and featuring in a wide variety of media from advertisements and cartoons to fiction and feature articles.^80^ Most notably of all, however, the Maxim gun became an emblem of late Victorian empire. The gun was officially adopted by the British military in 1887 and first saw significant action in the Anglo-Matabele war of 1893 where it was used to devastating effect, killing hundreds of King Lobengula’s lightly armed warriors and putting the rest, in the oddly understated words of one witness, ‘into a funk’.^81^ For some, such as the author of a pamphlet on *Our National Defences*, the Maxim’s ruthless efficiency was to be welcomed. Dismissing what he called ‘twaddle about honour and glory’ he noted with approval any mechanical contrivance which could ‘extinguish Mullahs in the Sudan [or] exterminate aborigines – for that’s what it comes to’.^82^ For opponents of empire, however, the Maxim gun epitomized the hypocrisy and violence attendant upon the spread of ‘civilization’ and the satirical press of the period carried frequent acid witticisms directed in particular at Cecil Rhodes’ wholehearted embrace of the weapon. *Punch*, for example, ran with the following quatrain entitled ‘To Bombastes [*sic*]’:

“Maxims of civilization?” That’s your fun.

Your only maxim is – a Maxim gun.

And “civilising”, in your cynic mirth,

Means – sweeping “niggers” off the face of the earth.^83^

More famous, and perhaps more elegant, is the closing refrain from Hilaire Bellloc’s 1898 poem ‘The Modern Traveller’ in which the aptly named ‘Blood’ reassures himself, in the midst of a native uprising: ‘Whatever happens we have got / The Maxim Gun, and they have not’.^84^

Yet even for the less overtly politically aligned, the spectre of the Maxim’s destructive power could be troubling. In 1899, for example, the ‘Greater Britain Exhibition’ was held at Earl’s Court, one of the highlights of which was ‘Mr .Frank Fillis’s Realistic Representation of Savage South Africa’. The culmination of this *tableau vivant* was a mock battle between Matabele warriors and British troops in which three Maxim guns ‘squirt[ed] out damage and death among the heathens’. A British officer having ‘observed triumphantly that the Matabele cannot stand the fire of the Maxim’, the reviewer for the journal *Pick*-*Me*-*Up* declared drily that ‘I do not … see anything to wonder at in that. I think I should be a little upset myself under the circumstances.^85^ Such sympathetic projections are similarly evident in this cartoon from *Funny Folks*, in which the mass destruction of the Matabele quite literally haunts the imagination of the English middle classes (Figure 3).

Even in military circles, reactions to the weapon were mixed. Ellis’ characterization of near-universal antipathy towards the new technology is a gross over exaggeration for many were clearly aware of its capacity to revolutionize both European and colonial warfare. It seems to have been particularly popular with those apt to embrace other cutting-edge technological innovations such as the bicycle, although many in the cavalry, the arm of service often derided as the most reactionary and conservative, were similarly convinced of its tactical value.^86^ Nonetheless, there were others who thought that such mechanization had a tendency to take the shine off military glory. Thus, for Winston Churchill slaughtering Dervishes during the Sudanese campaign of 1896–8 may have been ‘no less enjoyable than exciting’ but at heart it was all ‘a matter of machinery’ rather than heroic individualism.^87^ More worryingly, some believed that the machine gun constituted the ultimate deskilling of the British soldier and the final nail in the coffin of martial vigour. The soldier had now become little more than an industrial drone, possessing few skills or qualities beyond the ability to operate a weapon which required comparatively little input from him in any case. ‘We have been educating men to believe themselves invincible because of the superiority of arms’, claimed a speaker to the RUSI, ‘forgetting that when those scientific toys fail them, the men are powerless. Men should be taught to depend on their own individual strength, pluck and prowess and not in any single weapon’.^88^

The use of the term ‘toy’ here is revealing, for in debasing the martial qualities of a soldier already roundly dismissed as an underage weakling, the Maxim gun served to further undermine fragile masculine identities and to blur the boundary between men and boys. With its attractive combination of easy operation and sheer destructive capacity, the machine gun became an object of fascination for adolescent boys, and special features on the weapon regularly appeared in juvenile publications such as *Chums*. Likewise, by the turn of the century, toy machine guns were being marketed as part of a pleasure culture of war alongside other modern military technologies such as armoured trains (Figure 4).

Moreover, boys were also regularly pictured with the weapon, such as this image sent into *Heath and Home* of one Dunstan Salmon, dressed in a sailor’s suit, posing with (and judging from the spent casings in the foreground, perhaps even having fired) a lightweight 1895 version of the gun (Figure 5).^89^ This affinity between the Maxim gun and juvenility went beyond mere play. An early article on the gun which celebrated its perfection of the mechanistic principle was not alone in making the observation that the weapon was so efficient and self-regulating that it could literally ‘be kept firing by a boy’.^90^ By reducing the art of killing to the simple operation of a mechanical trigger, the Maxim gun, it seemed, was the ultimate weapon for the adolescent slum-bred ‘Tommy’ of the late Victorian imagination.

## The ‘Cult of the Bayonet’

Perhaps because of the range of uncomfortable associations it provoked, the cultural discourse of late nineteenth-century popular imperialism tended to obscure the figure of the machine gun as much as celebrate it. In Henry Newbolt’s famous poem, Vitaï Lampada (1892), for example, true masculinity can only find free reign when the ‘Gatling’s jammed’. Only then can its public-school-educated protagonist rally his men with the cry of ‘Play up! Play up! And play the game!’’^91^ Moreover, the spectre of mechanization and its ambiguous implications for masculine vigour can also be said to have played a key role in one of the most intriguing tactical developments of the period.

By the later 1880s not only had the Maxim gun been introduced into British military service, but the single-shot Martini Henry rifle had been replaced by the magazine-loading Lee Metford, followed in 1891 by the improved Lee Enfield. These weapons utilized a number of technological innovations which further transformed the face of battle. First, they fired smaller but more accurate bullets which were propelled by cordite, a smokeless low brisance explosive which did away with the clouds of smoke that once hung over the battlefield. Second, they allowed for up to ten rounds to be loaded into the weapon’s box magazine and fired sequentially by means of a handle-operated bolt.^92^ The soldier could thus engage targets at greater ranges, could more easily acquire and maintain those targets without being blinded by smoke or giving away his position, and could keep up a truly devastating rate of fire of between 20 and 30 aimed shots per minute.^93^

The results of such modern ‘arms of precision’ were clearly demonstrated at the Battle of Omdurman in 1898 when eleven thousand Dervish warriors were killed for the loss of only twenty-eight British and twenty Egyptian soldiers.^94^ Naturally, the devastating power of modern munitions led to a rethinking of conventional infantry tactics as European military theorists contemplated the potential impact of both combatant forces possessing equivalent firepower. Even the editor of *Chums* was moved to caution his overenthusiastic juvenile readers that ‘a great European war … would be too horrible to contemplate … Smokeless powder, Maxim guns, long range rifles – one might say that millions of deaths would be set down to these’.^95^ Many now believed the defender had gained the ascendency over the attacker and that any attempt to assault a well-defended position would result in significant, perhaps untenable, losses. Some theorists even went so far as to suggest that war, in its conventional form at least, was now all but impossible. Most notable among these was Ivan Bloch, a Polish banker-turned-futurologist whose portentous predictions gained a world-wide readership. The war of the near future, he prophesied, would be ‘a great war of entrenchments … The first thing every man will have to do, if he cares for his life at all, will be to dig a hole in the ground and throw up as strong an earthen rampart as he can to shield him from the hail of bullets which will fill the air’.^96^ Unable to break the strategic stalemate, the great powers of Europe would be consumed in an economic and industrial struggle that would eventually bankrupt them and lead to widespread famine. Effectively, he claimed, ‘the soldier, by natural evolution, has so perfected the mechanism of slaughter that he has practically secured his own extinction’.^97^

An integral part of Bloch’s theory was that the weapon that had so defined the nineteenth-century battlefield, the bayonet, was now a thing of the past. ‘In future’, he claimed, ‘war will be decided at ranges which will render the use of the bayonet impossible’.^98^ Other authorities concurred, suggesting that the bayonet was ‘The most ridiculous weapon known to modern warfare’. ‘In our modern battles’, they argued ‘the antagonists seldom come into actual contact. Battles are now fought with bullets instead of bayonets, and the latter, always a clumsy affair of doubtful utility, has become an altogether worthless incumbrance [*sic*]’.^99^

It might seem odd, therefore, that rather than receding into military history the bayonet actually reasserted itself with remarkable vigour at the turn of the twentieth century. There are, I would suggest, a number of reasons for this. First, in contrast to late nineteenth-century pessimism about the modern battlefield, early twentieth-century British military thinkers came increasingly to emphasize the importance of the offensive and of the offensive mind-set. Tim Travers calls this the ‘cult of the offensive’ and suggests that such strategy developed not in spite of modern fire-power but because of it; ‘just because fire-power now made the offensive very difficult’ he argues, ‘*therefore* the offensive must be heavily overemphasized’.^100^ Travers’ formulation is misleading, suggesting that such thinking was inherently irrational. In actual fact, late nineteenth- and early twentieth-century warfare provided numerous examples of the offensive triumphing over the defensive. Perhaps the most remarkable of these was the stunning victory of the Japanese over the Russians at Port Arthur in 1904. Although the Japanese had sustained extremely heavy casualties in the face of well-entrenched Russian troops they had eventually prevailed in large part due to their high morale and offensive spirit. Many Edwardian officers therefore argued for the continued necessity of élan in warfare and suggested that the British soldier should, like his Japanese counterpart, be conditioned to endure the terrifying effects of modern weapons and press on regardless.^101^

It is perhaps tempting to dismiss the resurgence of the bayonet as a reactionary phenomenon. However, if the offensive was truly to succeed then the bayonet was bound to prove a useful, if not necessarily a battle winning, weapon. What is more remarkable is the effect ascribed to it. Travers follows the line of contemporary military strategists in distinguishing between the ‘technological’ and the ‘psychological battlefield’.^102^ This period coincided with the rise of modern psychology and gave new impetus to conventional ideas such as morale. In this line of thinking the bayonet had an important psychological function, intimidating the enemy and emboldening those who wielded it in equal measure, turning men, as one later commentator put it, into ‘tigers’.^103^ Hence, despite all the technological innovations of the preceding decades, the individual soldier and his mental state had, by the early twentieth century, become the subject of intense interest and scrutiny among British military thinkers.

As well as improving the soldier’s mind, the bayonet was also held to improve his body. In his 1906 book *The Art of Attack*, the traveller and antiquarian Henry Swainson Cowper claimed that:

the personal strength of the warrior was an essential feature when all war was more or less hand to hand. … when we have sufficiently discounted all exaggerations, we may still believe that the hard open air life led by savages and medieval soldiery, coupled with the continued practice with weapons, did in reality produce a development of muscle, and a degree of personal skill, with which we have nothing to compare in civilized communities.^104^

As we have seen, there were many in military circles who would have agreed with Cowper’s assessment and lamented the physical disparity between the ‘savage’ warrior and the ‘civilized’ British soldier. Nonetheless, in spite of the increasing technological sophistication of modern munitions, the bayonet was held by some to represent a weapon which, if the object of ‘continued practice’ might yet develop that ‘muscle’ and ‘personal skill’ seemingly lacking in the average urban recruit. Thus it was that, in the late 1880s and early 1890s, the bayonet came to play a central role in British army training. The reissued *Army Drill Book* of 1889, for example, placed renewed emphasis on bayonet drill, while Lieutenant Colonel George Malcolm Fox of the Army Gymnastic Staff, who revolutionized the physical training of the army, put bayonet practice at the heart of his new regime. Indeed, in terms of sophistication and importance, bayonet training at the turn of the twentieth century surpassed anything seen at the height of its tactical importance in the early nineteenth. At Wellington Lines in Aldershot, Fox helped to develop elaborate ‘bayonet fencing’ techniques with the help of two Italian fencing masters while in 1890 Alfred Hutton published his *Fixed Bayonets*, a guide to bayonet fencing for the modern magazine rifle.^105^

Moreover, and as John Stone has argued, the value of the bayonet lay not simply in its tactical value and ‘technical characteristics’ but also in its social, cultural and emotional properties.^106^ In the case of Victorian Britain, the bayonet had come to act as a focal point for national mythologies of martial prowess. It was widely suggested that British victories in the Peninsula and Waterloo campaigns had been won at the point of the bayonet and that a fondness for ‘cold steel’ set the British soldier apart from his continental equivalent. Indeed, the bayonet was often celebrated in popular verse and song as the ultimate symbol of British imperial and military glory.^107^

What is also clear about the national mythologizing of the bayonet was that this weapon, a symbol of visceral martial aggression, was closely tied to visions of masculinity. In the aftermath of the battle of Abu Klea (1885), for example, a minor scandal erupted over the quality of bayonets supplied to the troops. According to one author, the’ want of confidence’ displayed by some of the soldiers could be put down to the fact that their bayonets were wrought with such poor steel that they were insufficiently sharp and ‘bent like a hoop iron without piercing the body of the Arabs’.^108^ Contemporary satirical representations of this combined failure of British industry and soldiery were suffused with an imagery of impotency that one does not have to be a Freudian to appreciate (Figure 6).

These factors, including the intimate association between the bayonet and popular conceptions of national manhood, help to explain why it was that, at precisely the moment when British martial masculinity was most under threat from the combined forces of mechanization and physical degeneration, the bayonet, previously dismissed by many theorists as tactically redundant, came into its own. Indeed, it is particularly ironic, given the reliance upon technology and the inability or unwillingness to engage the enemy in hand-to-hand combat that had so characterized previous imperial conflicts against lightly-armed native opponents, that this resurgence reached its peak in the Second South African war of 1899–1902, a conflict against an ethnically European opponent armed with weapons of equivalent, if not superior quality. In the face of the first truly modern war that the British army had fought and against the background of widespread concerns about national efficiency and the national physique, the bayonet gripped the public imagination, functioning as a kind of symbolic relief from the anxieties of *fin de siècle* modernity. One of the ‘Lessons of the War’, claimed the *County Gentleman*, was

that it is a fallacy that, with modern arms of precision in the hands of an enemy who knows how to use them, Tommy … can no longer get to close quarters with his foe, and use his bayonet with the same effect as in the great Duke’s days. And we have had demonstrated, what we have never doubted, although often told it was not so, that our gallant soldiers have lost none of the dash and dogged pluck which won the battle of Waterloo, Badajoz, and a hundred others.^109^

Even more sceptical commentators could not deny the imaginative potency of the weapon. One anonymous staff officer railed against what he called ‘the Cult of the Bayonet’, arguing that ‘the whole training of our infantry … is an encouragement of the erroneous idea … that all that has to be done is to get to the enemy’s position and that then the supreme moment arrives, and the bayonet makes the victory’. However, even he recognized that ‘The very phrase – “at the point of the bayonet” – expresses the national idea of force’, commenting that ‘no illustrated newspaper of today convinces its readers of the reality of the war scenes depicted, without a picture of a transfixed Boer’.^110^ A survey of the contemporary press reveals that such images were indeed remarkably common (Figure 7).

It is hard not to see the ‘cult of the bayonet’ as, in part, a reaction to late nineteenth-century anxieties about military masculinity, as an attempt to reassert the prowess of the individual soldier in the face of an increasingly mechanized and de-humanized world. Indeed, many attempts to proclaim the continued importance of the ‘Tommy’ and his bayonet were couched in a language which set man and machine in opposition. ‘The flesh and blood of the solider is not to be steeled or hardened by any mechanical process such as may be applied to plates or gun-barrels’, claimed one commentator. Nevertheless, he continued, while in the great war to come, ‘science on either side will neutralize itself, nerve, pluck, courage, whatever it may be called, will turn the scale’.^111^ When that war did eventually come, it is remarkable that, despite being the most technologically advanced conflict the world had ever seen, British military authorities continued to ‘fetishize’ the bayonet, with men such as Captain Leopold McLaglen developing elaborate bayonet fighting techniques and claiming that the war, far from rendering the bayonet redundant, had made it more important than ever.^112^

## Conclusion

In hindsight it is clear that early twentieth-century British military faith in the potency of the offensive was misplaced. So too was the belief that the individual soldier, steeled by martial spirit and clutching his bayonet-tipped rifle would win the day. Ultimately, élan and the bayonet were not enough to break the technologically enforced stalemate of the Western Front. Quick-firing artillery and the machine gun remained the kings of the battlefield and the answer to the deadlock, when it came, was perhaps the ultimate technological development of the war: the tank. Nonetheless, the bayonet continued to exert a powerful psychological grip over the British army. In many ways, the prospect of hand-to-hand combat, of ‘getting to grips’ with the enemy, served as a kind of emotional compensation for the sense of impotence engendered by the anonymity of death in an age of distance warfare. As Charles Pritchard Clayton recalled of his time as a British officer in the trenches:

There is a grim satisfaction in the fixing of bayonets. For long hours we have been baited, held at a disadvantage. One by one they have killed or hurt our comrades … “Come on Fritz” is the shout and a fellow near me mutters quietly, “I hope to God they come”.^113^

This article suggests that the significance of such weapons can only be fully understood by embedding them within their cultural context. Such an approach is in keeping with recent historiographical developments which have demonstrated the critical importance of masculinity in understanding the experience and conduct of the First World War.^114^ The bayonet, it maintains, functioned as a powerful, albeit ambivalent, expression of manhood, one that had been undermined and ultimately reasserted in the face of mechanization.

What this article also argues is that forms of technology such as the breech-loading rifle and machine gun were not simply passive objects. Their use, their reception and their representation were not value neutral. Rather, they were, in a profoundly Latourian sense, historical actors in their own right, capable of shaping social relations and social practises.^115^ The advent of these weapons may have held practical advantages. It may have helped secure the territorial and geopolitical gains of late Victorian imperialism. But it was far from unproblematic and could also be productive of anxiety and doubt, ethically, politically and, in the context of *fin de siècle* fears about degeneration, racially.

In this sense, the breech-loading rifle and the machine gun were powerful symbols of technological and cultural modernity. They promised much and delivered it too. But like modernity itself, they were ultimately far more complex and ambiguous than their superficial mechanical simplicity might suggest.

## Funding

This work was supported by the Wellcome Trust [grant number 108667/Z/15/Z].

## Notes on contributor

***Michael Brown*** is a Reader in History at the University of Roehampton and a Wellcome Trust Investigator in Medical Humanities. He has written widely on the history of nineteenth-century British medicine as well as the history of masculinity and gender. His publications include *Performing Medicine: Medical Culture and Identity in Provincial England c. 1760-1850* (Manchester University Press, 2011) as well as articles on medicine, masculinity and war (*Journal of British Studies*) the cultures of radical medical reform (*Social History* and *English Historical Review*) and surgery and empire (*Journal of Social History*).

## Disclosure statement

No potential conflict of interest was reported by the author.

## Notes

1. George Orwell, *The Road to Wigan Pier* (London: Penguin, 2001), pp. 90–1.

2. Daniel Pick draws together other associations between the abattoir and modern war in *War Machine: The Rationalisation of Slaughter in the Modern Age* (New Haven: Yale University Press, 1993), pp. 178–88.

3. Olive Anderson, ‘The Growth of Christian Militarism in mid-Victorian Britain’, *English Historical Review* vol. 86, No. 338 (1971), pp. 46–72; Anne Summers, ‘Militarism in Britain before the Great War’, *History Workshop Journal* vol. 2, No. 1 (1976), pp. 104–123.

4. Michael Howard, ‘Empire, War and Race in pre-1914 Britain’, in Hugh Lloyd-Jones, et. al (eds), *History and Imagination* (London: Holmes and Meier, 1981), p. 352.

5. John M MacKenzie, ‘Introduction: Popular Imperialism and the Military’, in John. M. MacKenzie (ed.), *Popular Imperialism and the Military* (Manchester: Manchester University Press, 1992), p. 9.

6. Michael Paris, *Warrior Nation: Images of War in British Popular Culture, 1850*–*2000* (London: Reaktion, 2000). See also Graham Dawson, *Soldier Heroes: British Adventure, Empire and the Imagining of Masculinities* (London: Routledge, 1994).

7. Summers, ‘Militarism in Britain’, p. 105.

8. MacKenzie, ‘Introduction’, p. 1.

9. Mary A. Conley, *From Jack Tar to Union Jack: Representing Naval Manhood in the British Empire, 1870*–*1918* (Manchester: Manchester University Press, 2009).

10. MacKenzie, ‘Introduction’, p. 3.

11. John Adam Cramb, *Reflections on the Origin and Destiny of Imperial Britain* (London: Macmillan, 1900); Paul Crook, *Darwinism, War and History: The Debate over the Biology of War from the ‘Origin of Species’ to the First World War* (Cambridge: Cambridge University Press, 1994).

12. Bernard Semmel, *Imperialism and Social Reform: English Social*-*Imperial Thought, 1895*–*1914* (Cambridge, MA: Harvard University Press, 1960).

13. Ignatius F. Clarke, *Voices Prophesyng War: Future Wars, 1763*-*3749*. 2nd ed. (Oxford: Oxford University Press, 1992); Crook, *Darwinism, War and History*, pp. 63–97.

14. John Ruskin, ’Lecture III: War’ in *The Crown of Wild Olive: Four Lectures on Industry and War* (Orpington: George Allen, 1889); MacKenzie, ‘Introduction’, 4–5.

15. Karl Pearson, *National Life from the Standpoint of Science* (London: Adam and Charles Black, 1905), p. 46; Howard, ‘Empire, War and Race’, p. 350.

16. Daniel Pick, *Faces of Degeneration: A European Disorder, c. 1848*–*1918* (Cambridge: Cambridge University Press, 1993); Anson Rabinbach, *The Human Motor: Energy, Fatigue, and the Origins of Modernity* (Berkeley: University of California Press, 1992).

17. Crook, *Darwinism, War and History*, p. 63.

18. Geoffrey Searle, *The Quest for National Efficiency: A Study in British Politics and Political Thought, 1889*–*1914* (Berkeley: University of California Press, 1971); idem, *Eugenics and Politics in Britain, 1900*–*1914* (Noordhoff: Kluwer, 1976).

19. Heather Streets, *Martial Races: The Military, Race and Masculinity in British Imperial Culture, 1857*–*1914* (Manchester: Manchester University Press, 2004), pp. 95–121.

20. Arnold White, *Efficiency and Empire* (London: Methuen and Co., 1901), pp. 95–121.

21. Daniel R. Headrick, *The Tools of Empire: Technology and European Imperialism in the Nineteenth Century* (Oxford: Oxford University Press, 1981); idem, *Power Over Peoples: Technology, Environments, and Western Imperialism, 1,400 to the Present* (Princeton: Princeton University Press, 2010).

22. The importance of industrial urbanism can be seen in the contrast often drawn between the enfeebled bodies of British soldiers and the physical hardiness of white Australians and New Zealanders or those of ‘marital races’ such as Sikhs, Gurkhas, etc. See Streets, *Martial Races*.

23. For the more optimistic view of British tactical innovation, see Howard Bales ‘Technology and Tactics in the British Army, 1866–1900’, in Roland Haycock and Keith Nelson (eds), *Men Machines and War* (Waterloo, ON: Wilfred Laurier University Press, 1988); idem., ‘Technology and Imperialism: A Case Study of the Victorian Army in Africa’, *Victorian Studies* vol. 24, No. 1 (1980), pp. 83–104; Stephen Beardsley, *Doctrine and Reform in the British Cavalry, 1880*–*1918* (Aldershot: Ashgate, 2008); Spencer Jones, *From Boer War to World War: Tactical Reform in the British Army, 1902*–*1914* (Oklahoma: University of Oklahoma Press, 2012). For the more pessimistic angle, see John Ellis, *A Social History of the Machine Gun* (Baltimore: Johns Hopkins University Press, 1976); Tim Travers, *The Killing Ground: The British Army, the Western Front and the Emergence of Modern Warfare, 1900*–*1918* (London: Routledge, 1993); Shelford Bidwell and Dominick Graham, *Fire*-*Power: The British Army Weapons and Theories of War, 1904*–*1945* (Barnsley: Pen and Sword Military Classics, 2004).

24. Paul Hodges, ‘They don’t like it up ’em!’: Bayonet fetishization in the British Army during the First World War’, *Journal of War and Culture* vol. 1, No. 2 (2008), pp. 123–138; John Stone, ‘The Point of the Bayonet’, *Technology and Culture* vol. 53, No. 4 (2012), pp. 885–908.

25. Leith Adams, ‘The Recruiting Question, Considered from a Military and a Medical Point of View’. *Journal of the Royal United Services Institute* vol. 18 (1875), p. 57.

26. Adams, ‘The Recruiting Question’, pp. 65,60.

27. Philip Henry, *Notes of Conversations with the Duke of Wellington, 1831*–*1851* (New York: Longmans, Green and Co., 1888), p. 14. As Matthew McCormack has shown, anxieties about the embodied qualities of the nation’s defenders had a significant prehistory linked to periods of political and military crisis. *Embodying the Militia in Georgian England* (Oxford: Oxford University Press, 2015).

28. Anon., *The Thinking Bayonet* (London: W. Mitchell and Co., 1871), p. 4.

29. Henry W. L. Hime, ‘Universal Conscription: The Only Answer to the Recruitment Question’, *Journal of the Royal United Services Institute* vol. 19 (1876), pp. 92 – 127; J. L. A. Simmons, ‘The Critical Condition of the Army’, *The Nineteenth Century* vol. 14, No. 77 (1883), pp. 165–188.

30. William F. Butler, ‘The New Army and the Old Test’, *The Fortnightly Review* vol. 33, No. 94 (1883), pp. 153–170.

31. David French argues that, in terms of recruitment, the creation of truly localized ‘regiments’ was generally unsuccessful and that regimental identities and traditions were generally fabricated. *Military Identities: The Regimental System, the British Army, and the British People c.1870*–*2000* (Oxford: Oxford University Press, 2005).

32. Frederick Stetson Allen, *The British Army 1860*–*1900: A Study of the Cardwell Manpower Reforms* (Boston: Harvard University Press, 1960); Edward Spiers, *The Late Victorian Army, 1868*–*1902* (Manchester: Manchester University Press, 1992), pp. 1–29; Brian Bond, ‘Edward Cardwell's Army Reforms, 1868–74’ in Harold E. Raugh (ed.), *The British Army, 1815*–*1914* (Aldershot: Ashgate, 2006).

33. Edward Sullivan, *The Froth and the Dregs* (London: Edward Stanford, 1871), pp. 75–6.

34. William Aitken, *The Growth of the Recruit and Young Soldier with a view to a judicious selection of ‘growing lads’ for the Army, and a regulated system of training for recruits*. 2nd ed. (London: Macmillan and Co, 1887).

35. Roberta J. Park, ‘Biological thought, athletics and the formation of a “man of character”: 1830–1900’, in J. A. Mangan and James Walvin (eds), *Manliness and Morality: Middle*-*Class Masculinity in Britain and America, 1800*-*1940* (Manchester: Manchester University Press); J. A. Mangan, ‘Athleticism: A Case Study of the Evolution of an Educational Ideology’, *International Journal of the History of Sport* vol. 27, nos 1–2 (2010), pp. 60–77.

36. Carolyn Steedman, ‘Bodies, Figures and Physiology: Margaret McMillan and the Late Nineteenth-Century Remaking of Working-Class Childhood’, in Roger Cooter (ed.), *In the Name of the Child: Health and Welfare, 1880*–*1940* (London: Routledge, 1992).

37. James Campbell, *‘The Army Isn’t All Work’: Physical Culture and the Evolution of the British Army, 1860*–*1920* (Farnham, Ashgate, 2012), pp. 23–44.

38. Sullivan, *The Froth*, p, 78.

39. Quoted in Sullivan, *The Froth*, p. 80.

40. Sullivan, *The Froth*, p. 78.

41. Hime, ‘Universal Conscription’, p. 105.

42. Frederick Roberts, ‘Free Trade in the Army’, *The Nineteenth Century* vol. 15, No. 88 (1884), pp. 1,055-74.

43. Adams, ‘The Recruiting Question’, pp. 56–7.

44. G. M. Onslow, ‘The Physique of the Solider and his Physical Training’*, Journal of the Royal United Services Institute* vol. 32 (1888–9), p. 727.

45. J. Moody, ‘Recruiting for her Majesty’s Service’, *Journal of the Royal United Services Institute* vol. 39 (1885), p. 569.

46. Hime, ‘Universal Conscription’, p. 107.

47. Hime, ‘Universal Conscription’, p. 106.

48. W. H. James, ‘On Some Changes in Tactics Caused by the Increasing Power of Modern Fire’, *Journal of the Royal United Services Institute* vol. 38 (1885), pp. 925–6; Headrick, *Tools of Empire*, pp. 96–103.

49. John A. English, *On Infantry* (New York: Praeger, 1981), pp.1–8.

50. Gervase Phillips, ‘Military Morality Transformed: Weapons and Soldiers on the Nineteenth-Century Battlefield’, *Journal of Interdisciplinary History* vol. 41, No. 4 (2011), pp. 565–90.

51. Anon., *The Thinking Bayonet*, p. 8.

52. Anon., *The Thinking Bayonet*, pp. 8–11.

53. Richard Wilbraham, ‘On the Causes which have led to the Pre-Eminence of Nations in War’, *Journal of the Royal United Services Institute* vol. 21 (1878), p. 484.

54. Saul David, *Zulu: The Heroism and Tragedy of the Zulu War of 1879* (London: Penguin, 2004); Ian Knight, *Brave Men’s Blood: The Epic of the Zulu War, 1879* (Barnsley: Pen and Sword Military Classics, 2005).

55. Harding Lawrence, ‘I’sandula!’, *Chambers Journal of Popular Literature, Science and Arts* vol. 799 (1879), p. 256; Michael Lieven, ‘Heroics and the Making of Heroes: The Anglo-Zulu War of 1879’, *Albion* vol. 30, No. 3 (1998), pp. 419–38.

56. In total, 11 Victoria Crosses were awarded to the defenders of Rorke’s Drift, the largest single award in British history.

57. Catherine Anderson, ‘Red Coats and Black Shields: Race and Masculinity in British Representations of the Anglo-Zulu War’, *Critical Survey* vol. 20, No. 3 (2008), pp. 6–28. ‘Zulus Vanquished and Zulus Victorious’, *Funny Folks* 17 May 1879, p. 155.

58. Bertram Mitford, *Through the Zulu Country: Its Battlefields and Its People* (London: Kegan Paul, Trench and Co., 1883); A. Wilmot, *History of the Zulu War* (London: Richardson and Best, 1880).

59. H. Knollys, ‘Some Very Noble Savages’. *Blackwoods Edinburgh Magazine* 149:907 (1891), p. 621. On the classically-inspired bodily aesthetics of the period, see Michael Anton Budd, *The Sculpture Machine: Physical Culture and Body Politics in the Age of Empire* (Basingstoke: Macmillan, 1997).

60. Anderson, ‘Red Coats’, pp. 21–2.

61. Quoted in Archibald Forbes, ‘The Recruiting Problem’, *The Nineteenth Century* vol. 29 No.169 (1891), p. 399.

62. Frederick Roberts, *Correspondence of General Sir Frederick Roberts, 1890*–*93. Vol. 2. Correspondence with India while Command*-*in*-*Chief in Madras, 1881*–*85*, British Library, IOR/L/MIL/17/5/1,615, p. 103.

63. This formation was traditionally used to defend against enemy cavalry.

64. ‘Desert Warfare: Being the Chronicle of the Eastern Soudan Campaign’, *Athenaeum* 19 July 1884, p.72; See also, ‘Our System of Infantry Tactics: What Is It?’, *Nineteenth Century* vol. 17, No. 99 (1885), pp. 836–7.

65. Garnett Wolseley, ‘The Negro as a Soldier’. *The Fortnightly Review* vol. 44, No. 264 (1888), p. 703. The contrast here with the attitude towards British ‘boy soldiers’ is intriguing.

66. Dawson, *Soldier Heroes*; Paris, *Warrior Nation*. See also, Jeffrey Richards (ed.), *Imperialism and Juvenile Literature* (Manchester: Manchester University Press, 1989); Bradley Deane, ‘Imperial Boyhood: Piracy and the Play Ethic’, *Victorian Studies* vol. 53, No. 4 (2011), pp. 689–711.

67. George Manville Fenn, ‘Off to the Wilds’, *The Union Jack*, 12 January 1882, p. 231.

68. Paris, *Warrior Nation*, p. 70.

69. Ellis, *Social History of the Machine Gun*, pp. 25–33. The Gatling gun was not technically a machine gun, in that it was not reloaded automatically but rather by human motive power. Nonetheless, it is widely considered as the forerunner to later fully mechanized developments.

70. Ellis, *Social History of the Machine Gun*, pp. 62, 70.

71. Ellis, *Social History of the Machine Gun*, pp. 63–4.

72. Ellis, *Social History of the Machine Gun*, pp. 79–109.

73. William Gardner, ‘Machine-Guns and How to Use Them’, *Journal of the Royal United Services Institute* vol. 26 (1883), p. 111. Nearly 500 Zulus are said to have been killed by Gatling guns at the battle of Ulundi. The total number of casualties was far higher. Ellis, *Social History of the Machine Gun*, p. 84.

74. E. Rogers, ‘The Gatling Gun; its place in tactics’, *Journal of the Royal United Services Institute* vol. 19 (1876), pp. 443–4.

75. Quoted in Ellis, *Social History of the Machine Gun*, p. 27.

76. Rogers, ‘The Gatling Gun’, p. 441.

77. R. H. Armitt, ‘Machine-Guns, their Use and Abuse’, *Journal of the Royal United Services Institute* vol. 30 (1886–7), p. 44.

78. Quoted in Charles W. D. Beresford, ‘Machine Guns in the Field’, *Journal of the Royal United Services Institute* vol. 28 (1885), p. 947.

79. ‘A Visit to a Famous Inventor’, *Chums*, 5 September 1894, p. 31.

80. Friedrich Kittler, *Gramophone, Film, Typewriter*, trans Geoffrey Winthrop-Young and Michael Wutz (Stanford: Stanford University Press, 1999), pp. 124–5, 191–2.

81. C. H. W. Donovan, *With the Victoria Column in Matabeleland* (Aldershot: Gale and Polden, 1894), p. 22; Ellis, *Social History of the Machine Gun*, pp. 90–1.

82. W. D. Gainsford, *Our National Defences: What is the Matter with our “Army”?* (London: G. Routledge, 1905), pp. 12, 23.

83. ‘To Bombastes’, *Punch*, 4 September 1897, p. 107.

84. Ellis, *Social History of the Machine*, p. 94.

85. ‘Through the Opera Glass: Greater British Exhibition’, *Pick*-*Me*-*Up*, 12 August 1899, p. 335.

86. ‘In Front with a Cyclist Corps’, *Chums*,26 July 1899, pp. 775–6; Wilkinson Shaw, *The Elements of Modern Tactics*, eighth edition (London: Kegan Paul, 1894), pp. 319–20.

87. Winston Churchill, *The River War: An Historical Account of the Reconquest of the Sudan* (London: Longmans, Green and Co., 1902), pp. 205, 273.

88. Armitt, ‘Machine-Guns, their Use and Abuse’, p. 41.

89. Given that the letter from Irene Salmon was addressed from Erith, site of the Maxim-Nordenfelt factory, and that Maxim himself lived in nearby Bexley, it is possible the Salmon family had privileged access to Maxim and his weapon.

90. ‘The Progress of Machinery’, *The Friendly Companion* 1 July 1885, p. 188.

91. Henry Newbolt, *Poems: New and Old*. London (John Murray, 1921), p. 95.

92. Headrick, *Tools of Empire*, pp. 98–9.

93. The record for the Lee Enfield of 38 shots a minute within a 24-inch target at 300 yards was said to have been set by Sergeant Instructor Alfred Snoxall in 1914.

94. Headrick, *Power Over Peoples*, pp*.* 275–6.

95. ‘The Editor to his Friends’, *Chums*, 5 February 1896, p. 380.

96. Ivan S. Bloch, *Modern Weapons and Modern War: Being an Abridgement of ‘The War of the Future and its Technological, Economic and Political Relations’ … with a Prefatory Conversation with the Author by W. T. Stead* (London: Grant Richards, 1900), p. xxxvii.

97. Bloch, *Modern Weapons*, p. xvii.

98. Bloch, *Modern Weapons*, p. xiv.

99. ‘No Bayonet Charges Now’, *The Halfpenny Marvel* vol. 32 (1898), p. 15.

100. Travers, *The Killing Ground*, p. 43.

101. Travers, *The Killing Ground*, pp. 37–55.

102. For an early example of such thinking in the military sphere, see William V. Herbert, ‘The Psychology of the Battlefield’, *Journal of the Royal United Services Institute* vol. 41 (1897), pp. 1,490-1,510.

103. Campbell, *‘The Army Isn’t All Work’*, p. 157.

104. H. S. Cowper, *The Art of Attack: Being a Study in the Development of Weapons and Appliances of Offence, from the Earliest Times to the Age of Gunpowder* (Ulverston: W. Holmes, 1906), pp. 22–3.

105. Campbell, *‘The Army Isn’t All Work’*, pp. 52–6; Alfred Hutton, *Fixed Bayonets: A Complete System of Fence for the British Magazine Rifle* (London: William Clowes and Sons, 1890).

106. Stone, ‘The Point of the Bayonet’, p. 885.

107.
*The British Bayonet, A Patriotic Song Dedicated to Captain Billington, by H.S.K. and Walter Maynard* (London: Cramer, Beale and Chappell, 1860); ‘The Bayonet’, *Moonshine* 23 January 1886.

108. ‘Desert Warfare’, p. 73.

109. ‘Lessons of the War’, *The County Gentleman*, 9 December 1899, p. 1,550.

110. ‘The War and the Drill Book’, *Contemporary Review* vol. 78 (1900), p. 204.

111. ‘Engines of Destruction’, *Saturday Review of Politics, Literature, Science and Art* 9 September 1899, p. 319.

112. Leopold McLaglen, *Bayonet Fighting for War* (London: Harrison and Sons, 1916. For the centrality of the bayonet in army physical training in the First World War, see Campbell, *‘The Army Isn’t All Work’*, chapter 11.

113. C. P. Clayton, *The Hungry One* (Llandysul: Gomer Press, 1978), pp. 28–9.

114. For example, see Joanna Bourke, *Dismembering the Male: Men’s Bodies, Britain, and the Great War* (Chicago: Chicago University Press, 1996); Jessica Meyer, *Men of War: Masculinity and the First World War in Britain*. (Basingstoke: Palgrave Macmillan, 2008); Michael Roper, *The Secret Battle: Emotional Survival in the Great War* (Manchester: Manchester University Press, 2009); Anthony Fletcher, ‘Patriotism, the Great War and the Decline of Victorian Manliness’, *History* vol. 88 No. 334 (2014), pp. 40–72.

115. Bruno Latour, *Reassembling the Social: An Introduction to Actor*-*Network Theory* (Oxford: Oxford University Press, 2005).

